# Supplementary material for: The risk factors for mortality of diabetic patients with severe COVID-19: A retrospective study of 167 severe COVID-19 cases in Wuhan
Source: PLoS One. 2020 Dec 31;15(12):e0243602. doi: 10.1371/journal.pone.0243602 (PMC7774835; doi:10.1371/journal.pone.0243602)
Supplement: S1 Table — (DOCX) [file pone.0243602.s001.docx]

**S1 Table.** **Demographics, clinical characteristics and treatment of patients**

| Characteristics | Total (n=167) | Non-survivor (n=109) | Survivor (n=58) | p value |
| --- | --- | --- | --- | --- |
| Demographics | | | | |
| Age median (IQR), y | 65.0(56.0-72.0) | 68.0(62.0-76.0) | 56.0(48.0-65.0) | <0.001 |
| Sex |  |  |  | 0.526 |
| Male | 109(65.3%) | 73(67.0%) | 36(62.1%) |  |
| Female | 58(34.7%) | 36(33.0%) | 22(37.9%) |  |
| Signs and symptoms | | | | |
| Fever | 148(88.6%) | 92(84.4%) | 56(96.6%) | 0.019 |
| Cough | 117(70.1%) | 76(69.7%) | 41(70.7%) | 0.897 |
| Dyspnea | 76(45.5%) | 57 (52.3%) | 19(32.8%) | 0.016 |
| Diarrhea | 31(18.6%) | 25(22.9%) | 6(10.3%) | 0.046 |
| Chest tightness | 23(13.8%) | 18(16.5%) | 5(8.6%) | 0.159 |
| Fatigue | 16(9.6%) | 14(12.8%) | 2(3.4%) | 0.050 |
| Confusion | 4(2.4%) | 4(3.7%) | 0 | 0.299 |
| Comorbidity | 125(74.9%) | 89(81.7%) | 36(62.1%) | 0.005 |
| Cardiovascular diseases | 79(47.3%) | 57 (52.3%) | 22(37.9%) | 0.077 |
| Endocrine system diseases | 60(35.9%) | 46(42.2%) | 14(24.1%) | 0.021 |
| Digestive system diseases | 19(11.4%) | 18(16.5%) | 1(1.7%) | 0.004 |
| Respiratory system diseases | 18(10.8%) | 13(11.9%) | 5(8.6%) | 0.512 |
| Nervous system diseases | 7(4.2%) | 6(5.5%) | 1(1.7%) | 0.666 |
| Kidney diseases | 3(1.8%) | 3(2.8%) | 0 | 0.552 |
| Treatment |  |  |  |  |
| Antibiotic therapy | 150(89.8%) | 109(100.0%) | 41(70.7%) | <0.001 |
| Antiviral therapy | 167(100.0%) | 109(100.0%) | 58(100.0%) | - |
| Glucocorticoids | 135(80.8%) | 109(100.0%) | 26(44.8%) | <0.001 |
| Non-IMV | 167(100.0%) | 109(100.0%) | 58(100.0%) | - |
| IMV | 92(55.1%) | 92(84.4%) | 0 | <0.001 |
| ECMO | 4(2.4%) | 4(3.7%) | 0 | 0.299 |
| CRRT | 23(13.8%) | 23(21.1%) | 0 | <0.001 |
| Onset of symptom to, median (IQR), d | | | | |
| Hospital admission | 10(7,14) | 10(7,15) | 10(7,13) | 0.201 |
| Death or discharge | 24(18-30) | 24(18-29) | 26(19-31) | 0.232 |

Data are n (%) and median (IQR). Endocrine diseases include diabetes(55,32.9%) and thyroid disease; Cardiovascular diseases include hypertension, coronary heart disease, myocardial infarction, stenting, etc; Digestive system diseases include cholecystitis, pancreatitis, gallstones, liver cysts, malignant tumors of the digestive system etc; Respiratory diseases include chronic obstructive pulmonary disease, emphysema, tuberculosis and other related diseases; Nervous system diseases include stroke and other related disorders. Kidney diseases include uremia, renal cysts, etc; Non-IMV: any oxygen therapy other than IMV; IMV: Invasive mechanical ventilation. ECMO: Extracorporeal Membrane Oxygenation; CRRT: continuous renal replacement therapy.
