# Supplementary material for: The risk factors for mortality of diabetic patients with severe COVID-19: A retrospective study of 167 severe COVID-19 cases in Wuhan
Source: PLoS One. 2020 Dec 31;15(12):e0243602. doi: 10.1371/journal.pone.0243602 (PMC7774835; doi:10.1371/journal.pone.0243602)
Supplement: S2 Table — (DOCX) [file pone.0243602.s002.docx]

**S2 Table. Cox regression analysis of risk factors for mortality among severe patients**

| **Characteristics** | **Univariable HR (95% CI)** | ***P* value** | **Multivariable HR (95% CI)** | ***P* value** |
| --- | --- | --- | --- | --- |
| **Demographics and clinical characteristics** | | | | |
| **Age, years** | 1.055  (1.037-1.073) | <0.001 | 1.058  (1.029-1.087) | <0.001 |
| **male sex (*vs* Female)** | 1.146  (0.768-1.710) | 0.505 | - | 0.488 |
| **Comorbidity present (*vs* not present)** | | | | |
| **Cardiovascular diseases** | 1.456  (0.997-2.126) | 0.052 | - | 0.108 |
| **Diabetes** | 2.050  (1.393-3.016) | <0.001 | 2.273  (1.153-4.116) | 0.003 |
| **Respiratory system diseases** | 1.298  (0.726-2.319) | 0.378 | - | 0.271 |
| **Laboratory findings** | | | | |
| **Leucocytes (× 10⁹/L)** | 1.085  (1.055-1.116) | <0.001 | - | 0.230 |
| **Neutrophils (× 10⁹/L)** | 1.092  (1.061-1.123) | <0.001 | 1.091  (1.037-1.148) | 0.001 |
| **Lymphocytes (× 10⁹/L)** | 0.183  (0.088-0.379) | <0.001 | 0.120  (0.033-0.431) | 0.001 |
| **Platelets (× 10⁹/L)** | 0.995  (0.993-0.998) | <0.001 | - | 0.928 |
| **D-dimer (µg/mL)** | 1.068  (1.045-1.091) | <0.001 | - | 0.091 |
| **Lactate dehydrogenase (U/L)** | 1.002  (1.001-1.002) | <0.001 | - | 0.179 |
| **Glucose(mmol/L; normal range 3.9–6.1)** | 1.075  (1.040-1.112) | <0.001 | 1.091  (1.021-1.166) | 0.010 |
| **Hypersensitive cardiac troponin (pg/mL)** | 1.000  (1.000-1.000) | 0.004 | - | 0.420 |
| **Hypersensitive C-reactive protein (mg/L; normal range 0.0–10.0)** | 1.004  (1.002-1.007) | <0.001 | - | 0.874 |
| **Interleukin-2**[**R**](javascript:;)**(U/mL)** | 1.000  (1.000-1.000) | <0.001 | 1.000  (0.999-1.000) | 0.031 |
| **Interleukin-8(pg/mL)** | 1.002  (1.001-1.003) | <0.001 | - | 0.703 |
| **Interleukin-10(pg/mL)** | 1.028  (1.016-1.039) | <0.001 | - | 0.353 |

HR=hazard ratio.
