# Supplementary material for: The risk factors for mortality of diabetic patients with severe COVID-19: A retrospective study of 167 severe COVID-19 cases in Wuhan
Source: PLoS One. 2020 Dec 31;15(12):e0243602. doi: 10.1371/journal.pone.0243602 (PMC7774835; doi:10.1371/journal.pone.0243602)
Supplement: S3 Table — (DOCX) [file pone.0243602.s003.docx]

| **S3 Table Laboratory results of patients with Corona Virus Disease 2019** | | | | | | |
| --- | --- | --- | --- | --- | --- | --- |
|  | **Survival(N=58)** | |  | **Non-Survival(N=109)** | |  |
| **N=109** | **Admission*** | **Terminal stage** | **p value** | **Admission*** | **Terminal stage** | **p value** |
| **Blood routine** |  |  |  |  |  |  |
| Leucocytes (× 10⁹ per L; normal range 3.5–9.5) | 5.3(3.9-6.9) | 5.5(4.7-6.8) | 0.251 | 9.7(6.1-13.3) | 12.4(6.7-18.5) | <0.001 |
| Neutrophils (× 10⁹ per L; normal range 1.8–6.3) | 3.9(2.7-5.6) | 3.4(2.8-3.9) | 0.012 | 8.8(5.4-12.4) | 11.7(6.6-16.9) | <0.001 |
| Lymphocytes (× 10⁹ per L; normal range 1.1–3.2) | 0.7(0.6-1.0) | 1.6(1.2-2.0) | <0.001 | 0.5(0.4-0.7) | 0.4(0.2-0.7) | 0.016 |
| Hemoglobin (g/L; normal range 130.0–175.0) | 132.5(122.8-141.3) | 127.0(118.0-136.0) | 0.001 | 126.0(115.0-144.0) | 104.0(82.0-123.0) | <0.001 |
| Platelets (× 10⁹ per L; normal range 125.0–350.0) | 182.0(158.0-301.8) | 224.0(174.0-294.0) | 0.104 | 162.0(108.0-224.0) | 48.0(29.0-83.0) | <0.001 |
| **Coagulation function** |  |  |  |  |  |  |
| Prothrombin time (s; normal range 11.5–14.5) | 14.2(13.6-15.2) | 12.8(12.3-13.1) | 0.066 | 15.5(14.5-17.9) | 18.1(16.2-21.2) | <0.001 |
| Activated partial thromboplastin time (s; normal range 29.0–42.0) | 40.7(37.8-46.1) | 35.3(33.3-36.9) | 0.465 | 39.4(35.7-45.6) | 46.0(41.0-63.9) | <0.001 |
| Fibrinogen（g/L; normal range 2.0-4.0） | 5.2(3.9-6.1) | 4.5(4.2-5.1) | 0.465 | 4.7 (3.0-6.4) | 3.9 (2.5-5.5) | 0.001 |
| D-dimer (µg/mL; normal range 0.0–0.5) | 0.8(0.4-1.5) | 0.8(0.3-2.1) | 0.068 | 6.4(1.7-21.0) | 7.4(2.7-21.0) | 0.638 |
| **Blood biochemistry** |  |  |  |  |  |  |
| Alanine aminotransferase (U/L; normal range0.0–41.0) | 27.0(14.0-48.3) | 29.5(22.0-42.0) | 0.515 | 30.0(20.0-44.8) | 24.0(16.0-68.0) | 0.124 |
| Aspartate aminotransferase (U/L; normal range0.0–40.0) | 32.0(20.0-55.3) | 21.0(17.0-27.3) | <0.001 | 44.0(29.3-68.5) | 46.0(26.5-133.5) | 0.245 |
| Total bilirubin (μmol/L; normal range 0.0–26.0) | 9.5(7.45-12.0) | 8.4(6.5-10.8) | 0.019 | 13.6(9.5-20.2) | 16.7(11.4-31.4) | 0.005 |
| Direct bilirubin (μmol/L; normal range0.0-8.0) | 4.6(3.6-6.1) | 3.2(2.5-3.9) | <0.001 | 6.4(4.6-10.9) | 10.5(6.6-23.5) | 0.001 |
| Albumin (g/L;normal range 35.0–52.0) | 34.5(30.7-39.3) | 37.6(34.5-39.6) | 0.002 | 29.9(26.9-33.5) | 26.1(23.1-30.5) | <0.001 |
| Globulin (g/L;normal range 20.0–35.0) | 34.9(31.3-37.3) | 29.4(26.8-32.7) | <0.001 | 35.5(31.4-39.6) | 31.4(26.8-35.7) | <0.001 |
| Lactate dehydrogenase (U/L; normal range 135.0–225.0) | 330.0(234.5-421.8) | 192.0(160.5-228.8) | <0.001 | 502.5(421.8-680.5) | 628.5(434.5-1002.5) | 0.001 |
| Serum creatinine (μmol/L; normal range 59.0–104.0) | 76.5(61.3-84.3) | 71.0(57.3-80.5) | 0.087 | 88.0(69.0-117.8) | 129.0(78.5-277.5) | <0.001 |
| BUN(mmol/L; normal range 3.1～8.0) | 4.0(3.4-5.1) | 4.2(3.5-5.0) | 0.778 | 9.2(5.9-15.9) | 17.8(10.5-28.7) | <0.001 |
| Glucose (mmol/L; normal range 3.9–6.1) | 6.6(5.8-8.4) | 5.6(5.0-6.6) | <0.001 | 8.8(6.7-13.6) | 10.8(7.8-17.8) | 0.010 |
| Hypersensitive cardiac troponin (pg/mL; normal range 0.0-34.2) | 4.3(2.4-6.6) | 1.9(1.9-3.9) | 0.001 | 45.7(14.1-323.4) | 148.0(35.1-571.2) | 0.249 |
| N-terminal pro-brain Natriuretic Peptide (pg/mL; normal range 0.0-247.0) | 129.5(83.3-326.5) | 66.5(35.8-142.0) | 0.010 | 1180.0(372.0-3591.0) | 5805.0(1671.5-12969.0) | <0.001 |
| **Infection-related biomarkers** |  |  |  |  |  |  |
| Hypersensitive C-reactive protein (mg/L; normal range 0.0–10.0) | 56.4(24.8-97.4) | 2.3(0.8-5.8) | <0.001 | 98.9(59.9-161.4) | 136.9(67.3-238.6) | 0.014 |
| Procalcitonin (ng/mL; normal range 0.02–0.05) | 0.06(0.04-0.18) | 0.04(0.02-0.05) | <0.001 | 0.21(0.11-1.01) | 2.52(0.63-9.63) | <0.001 |
| Serum ferritin (ug/L; normal range 30.0-400.0) | 725.9(477.0-1405.0) | 574.7(331.8-879.5) | <0.001 | 1645.7(993.9-2497.3) | 3063.8(1396.5-13738.5) | <0.001 |
| **Cytokines** |  |  |  |  |  |  |
| Interleukin -1β(pg/mL; normal range 0.0–5.0)* | 5.0(5.0-5.0) | 11.1(11.1-11.1) |  | 5.0(5.0-5.0) | 5.0(5.0-10.8) | 0.001 |
| Interleukin-2 [receptor](javascript:;) (U/mL; normal range 223-710) | 685.5(518.3-938.3) | 400.5(321.5-712.0) | 0.002 | 1147.0 (834.0-1662.0) | 1291.0(890.0-2328.0) | 0.004 |
| Interleukin-6(pg/mL; normal range 0.0–7.0) | 7.7(2.2-32.3) | 3.4(2.4-6.4) | 0.004 | 58.9 (28.4-165.4) | 192.3(69.4-2008.8) | <0.001 |
| Interleukin-8(pg/mL; normal range 0.0-62.0) | 10.8(8.1-22.3) | 10.3(6.2-13.2) | 0.038 | 28.4(15.8-82.4) | 168.0(44.4-950.0) | <0.001 |
| Interleukin-10(pg/mL; normal range 0.0-9.1)* | 5.0(5.0-9.2) | 8.3(8.3-8.3) |  | 12.0(8.0-20.3) | 17.3(9.3-89.2) | <0.001 |
| Tumor Necrosis Factor α (pg/mL; normal range 0.0-8.1) | 8.5(6.5-10.9) | 7.6(5.3-10.8) | 0.444 | 10.7(7.5-17.5) | 16.3(9.4-38.7) | <0.001 |

Data are median (IQR). p values were calculated by Mann-Whitney U test.

*Patients with normal level of interleukin-1β (lower than 5 pg/mL) were presented as “5 pg/mL”. Only one patient at terminal stage in survival group had been tested for interleukin-1β and interleukin-10.
