# Supplementary material for: The risk factors for mortality of diabetic patients with severe COVID-19: A retrospective study of 167 severe COVID-19 cases in Wuhan
Source: PLoS One. 2020 Dec 31;15(12):e0243602. doi: 10.1371/journal.pone.0243602 (PMC7774835; doi:10.1371/journal.pone.0243602)
Supplement: S4 Table — (DOCX) [file pone.0243602.s004.docx]

| **S4 Table Laboratory results of patients with Corona Virus Disease 2019 on admission** | | | | | | |
| --- | --- | --- | --- | --- | --- | --- |
|  | **Survival(N=58)** | |  | **Non-Survival(N=109)** | |  |
| **N=109** | **Diabetic(n=11)** | **Non-Diabetic(n=47)** | **p value** | **Diabetic(n=44)** | **Non-Diabetic(n=65)** | **p value** |
| **Blood routine** |  |  |  |  |  |  |
| Leucocytes (× 10⁹/L; normal range 3.5–9.5) | 5.6(3.3-6.7) | 5.2(3.8-6.9) | 0.891 | 10.1(7.8-14.7) | 9.3(5.5-12.8) | 0.061 |
| Neutrophils (× 10⁹/L; normal range 1.8–6.3) | 4.2(2.5-5.6) | 3.7(2.7-5.5) | 0.894 | 9.5(6.9-13.3) | 8.15(4.4-11.5) | 0.059 |
| Lymphocytes (× 10⁹/L; normal range 1.1–3.2) | 0.7(0.6-0.8) | 0.7(0.5-1.0) | 0.949 | 0.5(0.4-0.7) | 0.5(0.4-0.7) | 0.591 |
| Hemoglobin (g/L; normal range 130.0–175.0) | 133.0(113.0-140.0) | 132.0(123.0-143.0) | 0.642 | 125.5(116.2-140.0) | 129.0(110.2-145.0) | 0.897 |
| Platelets (× 10⁹/L; normal range 125.0–350.0) | 171.0(134.0-308.0) | 188.0(159.0-300.0) | 0.359 | 160.5(108.5-230.0) | 165.5(104.2-224.0) | 0.986 |
| **Coagulation function** |  |  |  |  |  |  |
| Prothrombin time (s; normal range 11.5–14.5) | 14.4(13.7-15.6) | 14.2(13.5-15.1) | 0.526 | 16.3(14.6-18.2) | 15.3(14.3-17.7) | 0.390 |
| Activated partial thromboplastin time (s; normal range 29.0–42.0) | 41.2(32.0-49.5) | 40.6(37.9-45.4) | 0.960 | 38.0(33.9-43.9) | 41.60(36.72-46.1) | 0.070 |
| Fibrinogen（g/L; normal range 2.0-4.0） | 5.8(3.8-5.9) | 5.0(3.9-6.2) | 0.810 | 4.2(2. 8-5.9) | 5.2(3.1-6.6) | 0.214 |
| D-dimer (µg/mL; normal range 0.0–0.5) | 1.0(0.4-1.5) | 0.7(0.4-1.6) | 0.508 | 8.9(2.3-21.0) | 4.3(1.6-21.0) | 0.455 |
| **Blood biochemistry** |  |  |  |  |  |  |
| Alanine aminotransferase (U/L; normal range0.0–41.0) | 21.0(11.0-35.0) | 27.0(15.0-52.0) | 0.343 | 30.5(19.0-42.8) | 29.5(21.0-46.5) | 0.761 |
| Aspartate aminotransferase (U/L; normal range0.0–40.0) | 22.0(18.0-48.0) | 36.0(21.0-56.0) | 0.184 | 43.5(30.0-69.3) | 44.5(29.0-68.0) | 0.887 |
| Total bilirubin (μmol/L; normal range 0.0–26.0) | 8.60(7.50-11.90) | 9.60(7.30-12.3) | 0.663 | 13.9(10.0-22.9) | 12.9(8.4-19.2) | 0.192 |
| Direct bilirubin (μmol/L; normal range0.0-8.0) | 4.60(3.60-5.30) | 4.60(3.50-6.20) | 0.818 | 6.7(4.7-12.5) | 6.3(4.4-10.3) | 0.324 |
| Albumin (g/L;normal range 35.0–52.0) | 30.9(27.8-35.7) | 35.0(31.9-39.4) | 0.097 | 29.4(24.6-31.8) | 31.0(28.1-34.2) | 0.013 |
| Globulin (g/L;normal range 20.0–35.0) | 37.0(28.9-39.3) | 34.6(31.3-37.0) | 0.607 | 35.8(31.4-39.7) | 35.5(31.5-39.4) | 0.689 |
| Lactate dehydrogenase (U/L; normal range 135.0–225.0) | 259.0(243.0-479.0) | 347.0(233.0-419.0) | 0.520 | 547.5(444.7-772.5) | 479.5(384.2-608.2) | 0.037 |
| Serum creatinine (μmol/L; normal range 59.0–104.0) | 77.0(49.0-79.0) | 76.0(63.0-87.0) | 0.309 | 89.5(72.3-122.5) | 87.5(67.0-116.0) | 0.682 |
| BUN(mmol/L; normal range 3.1～8.0) | 4.70(3.20-6.00) | 4.00(3.40-5.00) | 0.758 | 9.9(7.3-17.6) | 8.4(5.9-15.6) | 0.184 |
| Glucose (mmol/L; normal range 3.9–6.1) | 8.63(7.02-13.46) | 6.47(5.69-7.34) | 0.002 | 11.3(7.7-17.7) | 7.7(6.6-10.9) | 0.002 |
| Hypersensitive cardiac troponin (pg/mL; normal range 0.0-34.2) | 5.20(2.30-8.50) | 3.80(2.40-5.90) | 0.243 | 71.5(18.7-1072.2) | 31.2(12.1-159.3) | 0.070 |
| N-terminal pro-brain Natriuretic Peptide (pg/mL; normal range 0.0-247.0) | 175.5(66.5-642.7) | 120.0(83.7-323.5) | 0.541 | 1603.0(424.5-3654.5) | 913.5(333.5-3644.6) | 0.677 |
| **Infection-related biomarkers** |  |  |  |  |  |  |
| Hypersensitive C-reactive protein (mg/L; normal range 0.0–10.0) | 79.3(27.0-84.8) | 55.8(18.3-100.2) | 0.864 | 106.5(64.9-168.3) | 87.8(47.1-161.2) | 0.458 |
| Procalcitonin (ng/mL; normal range 0.02–0.05) | 0.06(0.04-0.59) | 0.06(0.04-0.18) | 0.817 | 0.38(0.14-1.32) | 0.18(0.09-0.64) | 0.066 |
| Serum ferritin (ug/L; normal range 30.0-400.0) | 781.7(582.7-1964.0) | 662.6(438.7-1270.2) | 0.177 | 1814.8(1034.1-2722.3) | 1351.0(915.2-2429.2) | 0.185 |
| **Cytokines** |  |  |  |  |  |  |
| Interleukin -1β(pg/mL; normal range 0.0–5.0)* | 5.0(5.0-5.0) | 5.0(5.0-5.0) | 0.832 | 5.0(5.0-5.0) | 5.0(5.0-5.0) | 0.794 |
| Interleukin-2 [receptor](javascript:;) (U/mL; normal range 223-710) | 677.0(595.0-1035.0) | 694.0(459.0-929.0) | 0.402 | 1163.0(946.5-1586.8) | 1128.0(762.0-1809.0) | 0.938 |
| Interleukin-6(pg/mL; normal range 0.0–7.0) | 22.2(2.1-90.0) | 5.7(2.2-30.2) | 0.205 | 68.0(35.6-220.4) | 57.9(16.8-130.9) | 0.169 |
| Interleukin-8(pg/mL; normal range 0.0-62.0) | 21.8(10.6-64.5) | 10.0(7.3-19.5) | 0.008 | 29.4(19.5-104.1) | 26.6(12.4-77.0) | 0.465 |
| Interleukin-10(pg/mL; normal range 0.0-9.1) | 7.8(5.0-11.9) | 5.0(5.0-8.3) | 0.300 | 13.5(9.3-19.9) | 10.8(5.7-20.7) | 0.212 |
| Tumor Necrosis Factor α (pg/mL; normal range 0.0-8.1) | 9.7(7.7-11.6) | 8.3(6.1-10.8) | 0.209 | 11.8(7.7-21.6) | 9.90(7.2-15.0) | 0.134 |

Data are median (IQR). p values were calculated by Mann-Whitney U test.

* Patient's interleukin-1β level is all lower than 5 pg/mL and it is uniformly expressed as “5 pg/mL”.
