# Supplementary material for: The risk factors for mortality of diabetic patients with severe COVID-19: A retrospective study of 167 severe COVID-19 cases in Wuhan
Source: PLoS One. 2020 Dec 31;15(12):e0243602. doi: 10.1371/journal.pone.0243602 (PMC7774835; doi:10.1371/journal.pone.0243602)
Supplement: Attachment — Submitted filename: PONE-D-20-21885 (Covid 19).pdf [file pone.0243602.s006.pdf]

PLOS ONE

The risk factors for mortality of diabetic patients with severe COVID-19: a retrospective study of 167 severe COVID-19 cases in Wuhan

--Manuscript Draft--

**Manuscript Number:**	PONE-D-20-21885
**Article Type:**	Research Article
**Full Title:**	The risk factors for mortality of diabetic patients with severe COVID-19: a retrospective study of 167 severe COVID-19 cases in Wuhan
**Short Title:**	Diabetic patients with severe COVID-19
**Corresponding Author:**	Donghua Zhang<br>Tongji Hospital, Tongji Medical College, Huazhong University of Science and Technology<br>Wuhan, Hubei CHINA
**Keywords:**	coronavirus disease 2019; diabetes; cytokine; mortality; hyperglycemia
**Abstract:**	Diabetes is one of the most common comorbidities in adult patients with coronavirus disease 2019 (COVID-19). This study aimed to analyze the mortality risk factors of diabetic patients with COVID-19. A total of 167 patients with severe COVID-19, including 55 diabetic patients and 112 nondiabetic patients at Tongji Hospital, Wuhan, China from January 28, 2020, to March 10, 2020, were collected. The laboratory, radiological, management information and medical history of them were retrospectively reviewed. Potential mortality risk factors in diabetic patients with COVID-19 were evaluated by the proportional hazard Cox model. The clinical information of 167 patients with severe COVID-19 was analyzed. The median age was 65.0 years. Approximately 32.9% of patients had diabetes. In total patients, older age, diabetes, lymphocyte count, neutrophil count and blood glucose were associated with increased risk of death. In diabetic patients, increased mortality was associated with decreased lymphocyte count (≤0.45×10^9/L, HR 0.196, 95% CI 0.049-0.781, P =0.021), lactate dehydrogenase >600 U/L (HR 8.010, 95% CI 1.540-41.670, P =0.013), hsCRP >90 mg/L (HR 4.551, 95% CI 1.472-14.070, P =0.009) and interleukin-10 >10 U/mL (HR 5.362, 95% CI 1.239-23.199, P =0.025). COVID-19 patients with diabetes had a poor prognosis, especially when they had two or more of the following abnormalities ($\chi2$=58.62, P <0.001): lymphocyte count was ≤0.45×10^9/L, lactate dehydrogenase was >600 U/L, hsCRP >90 mg/L and IL-10 was >10 U/mL. For diabetic patients with COVID-19, more attention should be paid to the dynamic monitoring of cytokine levels, and the control of hyperglycemia.
**Order of Authors:**	Yan Hui
	Zhiqiong Wang
	Xiwen Tong
	Yi Li
	Xia Mao
	Lifang Huang
	Donghua Zhang
**Additional Information:**	
**Question**	**Response**
**Financial Disclosure**<br><br>Enter a financial disclosure statement that describes the sources of funding for the work included in this submission. Review the submission guidelines for detailed	The funders had no role in study design, data collection and analysis, decision to publish, or preparation of the manuscript.

Powered by Editorial Manager® and ProduXion Manager® from Aries Systems Corporation

requirements. View published research articles from *PLOS ONE* for specific examples.<br><br>This statement is required for submission and **will appear in the published article** if the submission is accepted. Please make sure it is accurate.<br><br>**Unfunded studies**<br>Enter: *The author(s) received no specific funding for this work.*<br><br>**Funded studies**<br>Enter a statement with the following details:<br>• Initials of the authors who received each award<br>• Grant numbers awarded to each author<br>• The full name of each funder<br>• URL of each funder website<br>• Did the sponsors or funders play any role in the study design, data collection and analysis, decision to publish, or preparation of the manuscript?<br>• **NO** - Include this sentence at the end of your statement: *The funders had no role in study design, data collection and analysis, decision to publish, or preparation of the manuscript.*<br>• **YES** - Specify the role(s) played.<br><br>* typeset	
**Competing Interests**<br><br>Use the instructions below to enter a competing interest statement for this submission. On behalf of all authors, disclose any competing interests that could be perceived to bias this work—acknowledging all financial support and any other relevant financial or non-financial competing interests.<br><br>This statement **will appear in the published article** if the submission is accepted. Please make sure it is accurate. View published research articles from *PLOS ONE* for specific examples.	The authors have declared that no competing interests exist.

**NO authors have competing interests**<br><br>Enter: *The authors have declared that no competing interests exist.*<br><br>**Authors with competing interests**<br><br>Enter competing interest details beginning with this statement:<br><br>*I have read the journal's policy and the authors of this manuscript have the following competing interests: [insert competing interests here]*<br><br>* typeset	
**Ethics Statement**<br><br>Enter an ethics statement for this submission. This statement is required if the study involved:<br><br>• Human participants<br>• Human specimens or tissue<br>• Vertebrate animals or cephalopods<br>• Vertebrate embryos or tissues<br>• Field research<br><br>Write "N/A" if the submission does not require an ethics statement.<br><br>General guidance is provided below. Consult the submission guidelines for detailed instructions. **Make sure that all information entered here is included in the Methods section of the manuscript.**	This single-center, retrospective study was approved by the Ethics Committee of Tongji Hospital, Tongji Medical College, Huazhong University of Science and Technology (TJ-IRB20200316) and was consistent with the Declaration of Helsinki. Written informed consent was not obtained from the patients, as the Ethics Committee approved the application for exemption from obtaining informed consent.

Powered by Editorial Manager® and ProduXion Manager® from Aries Systems Corporation

**Format for specific study types**<br><br>**Human Subject Research (involving human participants and/or tissue)**<br>• Give the name of the institutional review board or ethics committee that approved the study<br>• Include the approval number and/or a statement indicating approval of this research<br>• Indicate the form of consent obtained (written/oral) or the reason that consent was not obtained (e.g. the data were analyzed anonymously)<br><br>**Animal Research (involving vertebrate animals, embryos or tissues)**<br>• Provide the name of the Institutional Animal Care and Use Committee (IACUC) or other relevant ethics board that reviewed the study protocol, and indicate whether they approved this research or granted a formal waiver of ethical approval<br>• Include an approval number if one was obtained<br>• If the study involved *non-human primates*, add *additional details* about animal welfare and steps taken to ameliorate suffering<br>• If anesthesia, euthanasia, or any kind of animal sacrifice is part of the study, include briefly which substances and/or methods were applied<br><br>**Field Research**<br><br>Include the following details if this study involves the collection of plant, animal, or other materials from a natural setting:<br>• Field permit number<br>• Name of the institution or relevant body that granted permission	
**Data Availability**<br><br>Authors are required to make all data underlying the findings described fully available, without restriction, and from the time of publication. PLOS allows rare exceptions to address legal and ethical concerns. See the PLOS Data Policy and FAQ for detailed information.	Yes - all data are fully available without restriction

A Data Availability Statement describing where the data can be found is required at submission. Your answers to this question constitute the Data Availability Statement and **will be published in the article**, if accepted.<br><br>**Important:** Stating 'data available on request from the author' is not sufficient. If your data are only available upon request, select 'No' for the first question and explain your exceptional situation in the text box.<br><br>Do the authors confirm that all data underlying the findings described in their manuscript are fully available without restriction?	
**Describe where the data may be found in full sentences. If you are copying our sample text, replace any instances of XXX with the appropriate details.**<br><br>• If the data are **held or will be held in a public repository**, include URLs, accession numbers or DOIs. If this information will only be available after acceptance, indicate this by ticking the box below. For example: *All XXX files are available from the XXX database (accession number(s) XXX, XXX.)*.<br>• If the data are all contained **within the manuscript and/or Supporting Information files**, enter the following: *All relevant data are within the manuscript and its Supporting Information files.*<br>• If neither of these applies but you are able to provide **details of access elsewhere**, with or without limitations, please do so. For example:<br><br>*Data cannot be shared publicly because of [XXX]. Data are available from the XXX Institutional Data Access / Ethics Committee (contact via XXX) for researchers who meet the criteria for access to confidential data.*<br><br>*The data underlying the results presented in the study are available from (include the name of the third party*	All relevant data are within the manuscript and its Supporting Information files.

and contact information or URL).<br>• This text is appropriate if the data are owned by a third party and authors do not have permission to share the data.<br><br>* typeset	
Additional data availability information:	Tick here if the URLs/accession numbers/DOIs will be available only after acceptance of the manuscript for publication so that we can ensure their inclusion before publication.

Powered by Editorial Manager® and ProduXion Manager® from Aries Systems Corporation

Manuscript Click here to access/download;Manuscript;manuscript.docx

The risk factors for mortality of diabetic patients with severe COVID-19: a retrospective study of 167 severe COVID-19 cases in Wuhan

Yan Hui, MD[1¶], Zhiqiong Wang, MD[1¶], Xiwen Tong, MD[1¶], Yi Li, MD[1¶], Xia Mao, PhD[1], Lifang Huang, PhD [1*], Donghua Zhang, MD[1*]

[1]Department of Hematology, Tongji Hospital, Tongji Medical College, Huazhong University of Science and Technology, Wuhan, Hubei, China

*Corresponding author

E-mail: zdh_62@126.com and E-mail: huanglifang627@163.com

[¶]Contributed equally

Abstract

Diabetes is one of the most common comorbidities in adult patients with coronavirus disease 2019 (COVID-19). This study aimed to analyze the mortality risk factors of diabetic patients with COVID-19. A total of 167 patients with severe COVID-19, including 55 diabetic patients and 112 nondiabetic patients at Tongji Hospital, Wuhan, China from January 28, 2020, to March 10, 2020, were collected. The laboratory, radiological, management information and medical history of them were retrospectively reviewed. Potential mortality risk factors in diabetic patients with COVID-19 were evaluated by the proportional hazard Cox model. The clinical information of 167 patients with severe COVID-19 was analyzed. The median age was 65.0 years. Approximately 32.9% of patients had diabetes. In total patients, older age, diabetes, lymphocyte count, neutrophil count and blood glucose were associated with increased risk of death. In diabetic patients, increased mortality was associated with decreased lymphocyte count ($\leqslant 0.45\times10^9$ /L, HR 0.196, 95% CI 0.049-0.781, *P*=0.021), lactate dehydrogenase >600 U/L (HR 8.010, 95% CI 1.540-41.670, *P*=0.013), hsCRP >90 mg/L (HR 4.551, 95% CI 1.472-14.070, *P*=0.009) and interleukin-10 >10 U/mL (HR 5.362, 95% CI 1.239-23.199, *P*=0.025). COVID-19 patients with diabetes had a poor prognosis, especially when they had two or more of the following abnormalities ($\chi2$=58.62, *P*<0.001): lymphocyte count was $\leqslant 0.45\times10^9$ /L, lactate dehydrogenase was >600 U/L, hsCRP was >90 mg/L and IL-10 was >10 U/mL. For diabetic patients with COVID-19, more attention should be paid to the dynamic monitoring of cytokine levels, and the control of hyperglycemia.

Introduction

In December 2019, the outbreak of coronavirus disease 2019 (COVID-19) caused by severe acute respiratory syndrome coronavirus 2 (SARS-CoV-2) first occurred in Wuhan, China, and quickly spread abroad. The clinical manifestations of SARS-CoV-2 closely resembled those of SARS, and infected patients may develop acute respiratory distress syndrome (ARDS) with a high likelihood of admission to the intensive care unit (ICU) and subsequent mortality. In these severe cases, comorbidities are common, especially diabetes [1-3].

Information about the details of clinical course of COVID-19 patients with diabetes has not yet been well described. Several published studies reported the risk factors associated with severity and mortality in patients with COVID-19 [1-3]. To our knowledge, no previous study has been performed on the definite outcomes in patients with diabetes.

Therefore, we aimed to compare 55 COVID-19 patients with diabetes and 112 patients without diabetes to summarize the differences in clinical and laboratory features. In addition, univariate and multivariate analyses were used to evaluate the potential risk factors associated with the mortality of diabetic patients with COVID-19.

Materials and methods

Patients

A total of 167 patients with severe COVID-19, including 55 diabetic patients and 112 nondiabetic patients at Tongji Hospital, Tongji Medical College, Huazhong University of Science and Technology, Wuhan, China from January 28, 2020, to March 10, 2020, were retrospectively reviewed. The patients were classified as severe according to the National Recommendations for the Diagnosis and Treatment of COVID-19 (7th edition) [4]. Tongji Hospital is one of the hospitals responsible for the central treatment of severe SARS-CoV-2-infected adult patients assigned by the government. All patients enrolled in the present study were diagnosed based on the National Recommendations for the Diagnosis and Treatment of

COVID-19 (7th edition) [4] and the World Health Organization (WHO) interim guidelines [5]. The present study was approved by the Ethics Committee of Tongji Hospital, Tongji Medical College, Huazhong University of Science and Technology and was consistent with the Declaration of Helsinki. Written informed consent was not obtained from the patients, as the Ethics Committee approved the application for exemption from obtaining informed consent.

Data collection

The laboratory, radiological, management information and medical history, including the comorbidities, symptoms and signs of patients, were obtained from the electronic medical records. All data were reviewed in detail by two experienced physicians (YL and LH). In addition, the dynamic changes in the results of the laboratory tests during hospitalization were monitored.

Laboratory testing for SARS-CoV-2

Throat swab samples were collected to extract SARS-CoV-2 ribonucleic acid (RNA) from patients suspected to have SARS-CoV-2 infection. The laboratory confirmation of SARS-CoV-2 was achieved through the concerted efforts of the Chinese Center for Disease Prevention and Control (CDC) and Wuhan Institute of Virology. The real-time reverse transcription-polymerase chain reaction (RT-PCR) assay and diagnostic criteria were performed in accordance with the protocol established by the WHO [5].

Statistical analysis

Continuous variables were expressed as the median and interquartile range (IQR) values. Categorical variables were described as counts and percentages. Continuous variables were compared using independent group *t*-tests when the data were normally distributed. Otherwise, the Mann-Whitney test was used. Categorical variables were compared by chi-square test or Fisher's exact test. The proportional hazard Cox model was used, and univariate models were fitted with a single candidate variable one at a time. We used X-tile software to identify cutoff points for evaluating potential mortality risk factors in diabetic patients with COVID-19 [6].

The subdistribution hazards ratio (SDHR) with a 95% confidence interval (95% CI) was reported. The overall survival (OS) was estimated using the Kaplan-Meier method and compared using the log-rank test. These statistical analyses were performed using SPSS software version 25.0. Statistical significance was defined as a two-sided *P*-value of less than 0.05.

Results

We included 167 inpatients with COVID-19 in the final analysis. A total of 109 patients died during hospitalization, and 58 patients were discharged. The median age of the 167 patients was 65.0 years, ranging from 26.0 years to 92.0 years, and an approximately 1.9:1.0 male-to-female ratio was found. Comorbidities were present in most patients, with hypertension (40.1%) being the most common comorbidity, followed by diabetes (32.9%) and coronary heart disease (19.2%). The most common symptoms were fever (88.6%) and cough (70.1%), followed by dyspnea (45.5%) and diarrhea (18.6%). All patients had bilateral infiltrates on the chest X-ray. The treatment information is shown in S1 Table. The median time from illness onset to discharge was 26.0 days (IQR 19.0–31.0), whereas the median time to death was 24.0 days (IQR 18.0–29.0) (S1 Table).

We compared the laboratory markers obtained at admission and discharge or death between survivors and nonsurvivors. Levels of interleukin-2 receptor (IL-2R), IL-6, hypersensitive C-reactive protein (hsCRP), lactate dehydrogenase (LDH) and serum ferritin were clearly elevated in nonsurvivors compared with survivors throughout the clinical course and increased as illness deterioration progressed. Levels of lymphocytes at admission declined clearly in nonsurvivors compared with survivors and continued to decrease until the terminal stage was reached (Fig

1).

In the univariate analysis, the odds of in-hospital death were higher in patients with diabetes than in patients without diabetes. Older age, lymphopenia, leukocytosis, neutrophilia, thrombocytopenia, hyperglycemia and elevated d-dimer, LDH, hsCRP, IL-8, and IL-10 were also associated with death. We included 167 patients with complete data for all variables in the multivariate Cox regression model. We found that older age, diabetes, hyperglycemia, lymphopenia and neutrophilia at admission were associated with increased risk of death (S2 Table).

The median age of diabetic patients was greater than nondiabetic patients (P=0.001). We compared the laboratory results on admission between diabetic patients (n=55) and nondiabetic patients (n=112). Compared with nondiabetic patients, diabetic patients had significantly higher leucocyte count (P=0.005), neutrophil count (P=0.004), levels of d-dimer (P=0.018), LDH (P=0.007), hs-cTnT (P<0.001), BNP (P=0.032), procalcitonin (P=0.005), serum ferritin (P=0.002), IL-6 (P=0.001), IL-10 (P=0.001) and TNF (P=0.014). Diabetic patients had lower albumin levels than nondiabetic patients (P<0.001). Increased levels of IL-2R at admission were observed in the majority of patients, but there was no significant difference between diabetic patients and nondiabetic patients (P=0.053) (Table 1).

Table 1. Demographic, laboratory findings and treatment of patients on admission.

Characteristics	Total (n=167)	Diabetic patient (n=55)	Non-diabetic patient (n=112)	*P* Value
**Demographics**				
Age median (IQR), y	65.0 (56.0-72.0)	71.0 (59.0-78.0）	64.0 (54.0-69.0）	0.001
Sex				0.467
Male	109(65.3%)	38(69.1%)	71(63.4%)	
Female	58(34.7%)	17(30.9%)	41(36.6%)	
**Laboratory findings**				
Leucocytes (× 10^9/L; normal range 3.5 – 9.5)	7.6(5.1-11.4)	9.1(6.2-13.4)	6.8(4.6-10.7)	0.005
Neutrophils (× 10^9/ L; normal range 1.8 – 6.3)	6.3(3.8-10.2)	7.9(5.4-12.6)	5.4(3.4-9.6)	0.004
Lymphocytes (× 10^9 /L; normal range 1.1–3.2)	0.6(0.4-0.8)	0.6(0.5-0.7)	0.6(0.4-0.9)	0.153
D-dimer (μg/mL; normal range 0.0–0.5)	2.5(0.9-17.9)	3.9(1.2-21.0)	1.8(0.8-9.7)	0.016
Albumin (g/L; normal range 35.0–52.0)	31.5(27.9-35.0)	29.8(25.3-32.6)	32.5(29.5-35.3)	<0.001
Lactate dehydrogenase (U/L; normal range 135.0–225.0)	465.5(338.0-597.5)	504.0(347.0-708.0)	427.0(325.0-529.0)	0.007
Glucose (mmol/L; normal range 3.9–6.1)	7.7(6.3-11.8)	10.6(7.4-16.9)	6.9(6.1-9.4)	<0.001
Hypersensitive cardiac troponin (pg/mL; normal range 0.0-34.2)	14.4(4.7-121.9)	41.5(8.6-557.5)	10.3(3.8-59.9)	<0.001
N-terminal pro-brain Natriuretic Peptide (pg/mL; normal range 0.0-247.0)	504.0(161.5-1895.0)	790.0(199.0-2636.0)	353.5(126.3-1546.0)	0.032
Procalcitonin (ng/mL; normal range 0.02–0.05)	0.16(0.07-0.59)	0.23(0.10-1.18)	0.14(0.05-0.37)	0.005
Serum ferritin (ug/L; normal range 30.0-400.0)	1217.4(613.2-2006.6)	1715.8(833.4-2429.3)	1072.4(538.9-1724.4)	0.002
Interleukin -1β(pg/mL; normal range 0.0–5.0)	5.0(5.0-5.0)	5.0(5.0-5.0)	5.0(5.0-5.0)	0.729

Interleukin-2 receptor (U/mL; normal range 223-710)	954.0(643.5-1364.0)	1083.0(677.0-1501.0)	923.5(597.8-1311.3)	0.053
Interleukin-6(pg/mL; normal range 0.0–7.0)	36.3(9.5-96.4)	56.1(24.8-145.3)	25.9(5.3-68.1)	0.001
Interleukin-8(pg/mL; normal range 0.0-62.0)	21.9(10.5-47.3)	28.3(15.8-82.4)	16.0(9.5-36.3)	0.001
Interleukin-10(pg/mL; normal range 0.0-9.1)	9.0(5.0-15.9)	11.5(7.9-19.4)	6.7(5.0-12.6)	0.001
Tumor Necrosis Factor α (pg/mL; normal range 0.0-8.1)	9.4(7.2-14.3)	11.1(7.7-20.6)	9.1(6.8-13.2)	0.014
**Treatments**				
Antibiotic therapy	150(89.8%)	54(98.2%)	96(85.7%)	0.012
Antiviral therapy	167(100%)	55(100.0%)	112(100.0%)	-
Glucocorticoids	135(80.8%)	48(87.3%)	87(77.7%)	0.191
Non-IMV	167(100.0%)	55(100.0%)	112(100.0%)	-
IMV	92(55.1%)	32(58.2%)	60(53.6%)	0.573
ECMO	4(2.4%)	1(1.8%)	3(2.7%)	1.000
CRRT	23(13.8%)	4(7.3%)	19(17.0%)	0.088
**Outcomes**				
Survival	58(34.7%)	11(20.0%)	47(42.0%)	0.005
Died	109(65.3%)	44(80.0%)	65(58.0%)	

Data are median (IQR). p values were calculated by Mann-Whitney U test, χ^2 test, or Fisher's exact test, as appropriate. Non-IMV: any oxygen therapy other than IMV; IMV: Invasive mechanical ventilation. ECMO: Extracorporeal Membrane Oxygenation; CRRT: continuous renal replacement therapy.

We compared the laboratory results on admission between nonsurvivors and survivors in diabetic patients and non-diabetic patients. In diabetic patients (n=55), compared with survivors (n=11), nonsurvivors (n=44) had significantly increased leucocyte count (*P*<0.001), neutrophil count (*P*<0.001), levels of d-dimer (*P*<0.001), LDH (*P*<0.001), hs-cTnT (*P*<0.001), BNP (*P*=0.001), procalcitonin (*P*=0.006), serum ferritin (*P*=0.044), IL-2R (*P*=0.023), IL-6 (*P*=0.006) and IL-10 (*P*=0.023). Nonsurvivors with diabetes had lower lymphocyte count than survivors (*P*=0.006). Although blood glucose levels in both surviving and nonsurviving diabetic patients were greater than normal, there was no significant difference between the two groups (*P*=0.265).

Notably, among the nondiabetic patients, the blood glucose of the nonsurvivors was significantly higher than that of the survivors (*P*<0.001). In nondiabetic patients (n=112), compared with survivors (n=47), nonsurvivors (n=65) had significantly decreased level of albumin (*P*<0.001) and increased levels of IL-8 (*P*<0.001) and TNF-α (*P*=0.045) (Table 2).

Table 2. Demographic and laboratory findings in patients with COVID-2019 on admission.

**Characteristics**	**Diabetic patient (n=55)**			**Non-diabetic patient(n=112)**		
	Non-survivor (n=44)	Survivor (n=11)	*P* Value	Non-survivor (n=65)	Survivor (n=47)	*P* Value
**Demographics**						
Age median (IQR), y	72.0 (63.0-78.0）	57.0 (54.0-72.0)	0.016	66.0 (61.0-73.0)	55.0 (46.0-65.0）	<0.001
Sex			0.722			0.752
Male	31 (70.5%)	7 (63.6%)		42 (64.6%)	29 (61.7%)	
Female	13 (29.5%)	4 (36.4%)		23 (35.4%)	18 (38.3%)	
**Laboratory findings**						
Leucocytes (×10^9/L; normal range 3.5－9.5)	10.1 (7.9-14.7)	5.6 (3.3-6.8)	<0.001	9.4 (5.5-12.8)	5.2 (3.9-6.9)	<0.001
Neutrophils (×10^9/L; normal range 1.8－6.3)	9.5 (6.9-13.4)	4.2 (2.6-5.7)	<0.001	8.1 (4.5-11.5)	3.8 (2.7-5.6)	<0.001
Lymphocytes (×10^9/L; normal range 1.1–3.2)	0.5 (0.4-0.7)	0.7 (0.7-0.9)	0.006	0.5 (0.4-0.7)	0.8 (0.6-1.0)	<0.001
D-dimer (μg/mL; normal range 0.0–0.5)	8.9 (2.3-21.0)	1.0 (0.4-1.5)	<0.001	4.3(1.6-21.0)	0.7 (0.4-1.6)	<0.001
Albumin (g/L; normal range 35.0–52.0)	29.4 (24.6-31.7)	30.9 (27.8-35.7)	0.058	31.0 (28.7-34.2)	35.0 (31.9-39.4)	<0.001
Lactate dehydrogenase (U/L; normal range 135.0–225.0)	547.5 (444.8-772.5)	259.0 (243.0-479.0)	<0.001	479.0 (384.3-608.3)	347.0 (233.0-419.0)	<0.001
Glucose (mmol/L; normal range 3.9–6.1)	11.3 (7.7-17.7)	8.6 (7.0-13.5)	0.265	7.7 (6.6-10.9)	6.5 (5.7-7.3)	<0.001
Hyper-sensitive cardiac troponin	71.4 (18.7-1072.2)	5.2 (2.3-8.5)	<0.001	31.2 (12.1-159.3)	3.8 (2.4-5.9)	<0.001

(pg/mL; normal range 0.0-34.2)						
N-terminal pro-brain Natriuretic Peptide (pg/mL; normal range 0.0-247.0)	1603.0 (424.5-3654.5.0)	175.5 (66.5-642.8)	0.001	913.5 (333.5-3644.8)	120.0 (83.8-323.5)	<0.001
Procalcitonin (ng/mL; normal range 0.02–0.05)	0.39 (0.14-1.33)	0.06 (0.04-0.59)	0.006	0.18 (0.09-0.64)	0.06 (0.04-0.18)	<0.001
Serum ferritin (ug/L; normal range 30.0-400.0)	1814.7 (1034.1-2722.3)	781.7 (582.7-1964.0)	0.044	1351.0 (915.2-2429.2)	662.7 (438.8-1270.2)	<0.001
Interleukin -1β(pg/mL; normal range 0.0–5.0)	5.0 (5.0-5.0)	5.0 (5.0-5.0)	0.844	5 (5-5)	5 (5-5)	0.263
Interleukin-2 receptor (U/mL; normal range 223-710)	1163.0 (946.5-1586.7)	677.0 (595.0-1035.0)	0.023	1128.0 (762.0-1809.0)	694.0 (459.0-929.0)	<0.001
Interleukin-6(pg/mL; normal range 0.0–7.0)	68.0 (35.6-220.4)	22.2 (2.2-90.0)	0.006	57.9 (16.8-130.9)	5.8 (2.3-30.3)	<0.001
Interleukin-8(pg/mL; normal range 0.0-62.0)	29.4 (19.5-104.1)	21.8 (10.6-64.5)	0.169	26.6 (12.4-77.0)	10.0 (7.3-19.5)	<0.001
Interleukin-10(pg/mL; normal range 0.0-9.1)	13.5 (9.3-19.9)	7.8 (5.0-11.9)	0.023	10.8 (5.7-20.7)	5.0 (5.0-8.3)	<0.001
Tumor Necrosis Factor α (pg/mL; normal range 0.0-8.1)	11.8 (7.7-21.6)	9.7 (7.7-11.6)	0.236	9.9 (7.2-15.0)	8.3 (6.1-10.8)	0.045

Data are median (IQR). p values were calculated by Mann-Whitney U test.

In the univariate analysis, the odds of in-hospital death were higher in diabetic patients with

lymphopenia than in diabetic patients without lymphopenia. Older age, neutrophilia, thrombocytopenia, hyperglycemia and elevated LDH, serum creatinine, BUN, hs-cTnT, BNP, PCT, hsCRP, IL-6, IL-10 and TNFα were also associated with death. We included meaningful indicators from the univariate analysis of 55 diabetic patients in the multivariate Cox regression model. We found that lymphocyte count $>0.45\times10^9$ /L was a protective factor associated with survival in diabetic patients. LDH >600 U/L, hsCRP >90 mg/L and IL-10 >10 U/mL increased mortality (Table 3).

Table 3. Cox regression analysis of risk factors for mortality of diabetic patients with COVID-19.

Characteristics	Univariable HR (95% CI)	*P* value	Multivariable HR (95% CI)	*P* value
Age, years				
≤70	1(ref)			
>70	1.891 (1.034-3.456)	0.039	1.465 (0.522-4.115)	0.469
Neutrophils (× 10^9 /L)				
≤7	1(ref)			
>7	3.194 (1.586-6.429)	0.001	0.521 (0.121-2.247)	0.382
Lymphocytes (× 10^9 /L)				
≤0.45	1(ref)			
>0.45	0.316 (0.161-0.621)	0.001	0.196 (0.049-0.781)	0.021
Platelets (× 10^9 /L)				
≤65	1(ref)			
>65	0.351 (0.152-0.815)	0.015	2.334 (0.530-10.285)	0.263
Lactate dehydrogenase (U/L)				
≤600	1(ref)			
>600	2.919 (1.542-5.527)	0.001	8.010 (1.540-41.670)	0.013
Serum creatinine (μmol/L)				
≤85	1(ref)			
>85	3.082 (1.667-5.701)	<0.001	1.672 (0.379-7.369)	0.497
BUN (mmol/L)				
≤10	1(ref)			
>10	5.674 (2.791-11.536)	<0.001	0.769 (0.126-4.686)	0.776
Hypersensitive cardiac troponin (pg/mL)				
≤100	1(ref)			
>100	2.286 (1.243-4.206)	0.008	1.176 (0.345-4.010)	0.795
N-terminal pro-brain Natriuretic Peptide (pg/mL)				
≤1500	1(ref)			
>1500	3.379 (1.757-6.498)	<0.001	1.798 (0.426-7.591)	0.424
Procalcitonin (ng/mL)				
≤0.15	1(ref)			
>0.15	2.333 (1.202-4.527)	0.012	0.469 (0.124-1.775)	0.265

Hypersensitive C-reactive protein I (mg/L)				
≤90	1(ref)			
>90	2.200 (1.199-4.038)	0.011	4.551 (1.472-14.070)	0.009
Interleukin-2 receptor (U/mL)				
≤1000	1(ref)			
>1000	1.735 (0.927-3.246)	0.085	-	-
Interleukin-6 (U/mL)				
≤120	1(ref)			
>120	2.313 (1.185-4.514)	0.014	1.017 (0.218-4.755)	0.983
Interleukin-10 (U/mL)				
≤10	1(ref)			
>10	3.088 (1.489-6.403)	0.002	5.362 (1.239-23.199)	0.025
Tumor Necrosis Factor α (pg/mL)				
≤20	1(ref)			
>20	2.198 (1.083-4.461)	0.029	0.391 (0.092-1.651)	0.201

HR=hazard ratio.

Diabetic patients had higher mortality rate than nondiabetic patients (80.0% vs 58.0%, P=0.005) (Table 2). There was a significant difference in overall survival (OS) between diabetic patients and nondiabetic patients (*P*<0.001). This finding suggests that diabetes is a risk factor for reduced survival in severe cases. According to the multivariate cox regression model, four laboratory results affecting the survival of diabetic patients were calculated (lymphocyte count ≤0.45× 10^9 /L, CRP >90 mg/L, LDH >600 U/L, IL-10 >10 U/mL) (Table 3). The diabetic patients were divided into 5 groups according to the number of abnormal items, with significant differences in OS in the 5 groups (*P*<0.001) (Fig 2).

Discussion

In this single-center, retrospective and observational study, we enrolled 167 patients with severe COVID-19, with a median age of 65.0 years (IQR 56.0-72.0). Through the comparison of survivors (65.27%) and nonsurvivors (34.73%), it was found that there was a significant difference in the proportion of patients with endocrine diseases and digestive system diseases. Through further COX regression, univariate and multivariate analyses showed that older age, diabetes, neutrophil count, lymphocyte count and blood glucose were independent prognostic factors in patients with COVID-19. Diabetes was the only concomitant disease that affected prognosis.

Individuals with diabetes are at risk of severe infections, especially influenza and pneumonia [7]. Diabetes was an important risk factor for exacerbation or death after infection in Middle East Respiratory Syndrome Coronavirus (MERS-CoV) studies (8-10). In a severe acute respiratory syndrome (SARS) study, diabetes history and hyperglycemia were found to be independent predictors of death and morbidity [11]. At present, the researches on the prognosis of patients with diabetes and SARS-CoV-2 is limited and contradictory. In a study of 140 patients with COVID-19, 58.5% of the patients were nonsevere and only 12.1% of which had diabetes, suggesting that diabetes had no adverse effects in patients with mild disease [12]. However, a report on 72314 COVID-19 cases analyzed by the Chinese Centre for Disease Control and Prevention showed that the mortality rate of people with diabetes is increased [7]. The results of our study also suggested that diabetes was an independent prognostic factor for COVID-19 patients. In our study, the survival rate of diabetic patients was lower, and the time from attack to death was shorter than nondiabetic patients. Besides, in the study of SARS, it was found that viruses could enter the islet through combining to angiotensin converting

enzyme 2 (ACE2), damage the pancreatic β-cells and cause acute diabetes [13]. ACE2 was expressed in various tissues and organs of the human body. Significant ACE2/Ang[1-7]/Mas signaling pathway inhibition and increased ACE/AngII/AT1R pathway activity occur after SARS-CoV-2 bind to ACE2 in vivo [14]. The mortality occurs through the imbalance of the renin-angiotensin system (RAS) and an increase in the level of inflammatory factors[14]. It was shown that ACE2 was also the receptor of SARS-CoV-2 [12,15,16], therefore SARS-CoV-2 may also directly damage the pancreas through ACE2. According to the comparative analysis of survivors and nonsurvivors, it was found that in nondiabetic patients, the blood glucose of nonsurvivors was also significantly higher than survivors, indicating that even if there was no history of diabetes, hyperglycemia was also an adverse prognostic factor.

Through further research, compared with nondiabetic patients, levels of cardiac injury indexes (cardiac troponin, BNP), inflammation related indexes (PCT, ferritin), coagulation function (d-dimer), cytokines, and LDH markedly elevated in diabetic patients. Besides, albumin decreased significantly in diabetes, indicating that the basic condition of diabetic patients was poor. We further analyzed the prognosis of patients with diabetes and determined their cutoff points to make these prognostic factors specific, and then verified these factors through Kaplan-Meier method. Eventually, the result showed that lymphocyte count $\leq 0.45\times10^{9}$ /L, LDH >600 U/L, hsCRP >90 mg/L and IL-10 >10 U/mL were associated with increased risk of death.

Lymphocytopenia is a common characteristic in COVID-19 patients [17], and lymphocyte count $\leq 0.45\times10^{9}$/L was an independent prognostic factor for patients with diabetes in this study. Researches have shown that the immune cells of people and mice with type 2 diabetes had a

transition from regulatory T cells (Treg) or anti-inflammatory macrophages to proinflammatory macrophages and Th1 and Th17CD4+T cells [18,19]. The increase of LDH and CRP indicated that the injury of the body was severe and may be complicated with bacterial infection which affected the prognosis.

Acute lung injury (ALI) is a common consequence of a cytokine storm in the lung alveolar environment and circulatory system and is most commonly associated with suspected or proven infections in the lungs or other organs [20]. Pathogen-induced lung injury can progress into ALI or its more severe form, ARDS, as seen with SARS and influenza virus infections. In patients with SARS, pro-inflammatory factors (IL-1β, IL-6, IL-12, interferon-γ, inducible protein-10, monocyte chemoattractant protein-1) increased [21]. Cytokine storms are known to play an important role in the acute lung injury of patients with severe COVID-19 [22]. The IL-6 and IL-8 levels were markedly increased, consistent with the severity of the disease. However, compared with the survivors, IL-10 also increased significantly in our study, and the mortality of patients with severe COVID-19 complicated with diabetes was increased when IL-10 >10 U/mL. The relation between IL-10 and diabetes needs further research. Moreover, the level of cytokines in patients with diabetes was higher than nondiabetes, suggesting that diabetic patients were more likely to experience cytokine storms, thus aggravating the lung damage. Twenty-three patients (including four patients with diabetes) with COVID-19 received continuous renal replacement therapy (CRRT) treatment but eventually died, indicating that it may be too late to use CRRT at these patients.

In this study, the age of the nonsurvivors was significantly higher than survivors in both diabetic and nondiabetic patients, and the univariate and multivariate results were consistent.

As age increases, the underlying inflammation and immune system disorders associated with aging may be related to the reactivation of latent viral infections and the release of endogenous damage-related pattern recognition receptor (PRR) ligands [23]. Additionally, there are more complications in the elderly. Therefore, we should be particularly vigilant against the progress of the disease and implement the necessary treatment promptly for elderly patients.

133 (79.6%) patients received glucocorticoid treatment in this study, of which 47 cases were complicated with diabetes. Inflammation and cytokine storms accelerate the progression of the disease in patients with severe disease symptoms. It is recommended that glucocorticoids could be used for a short period time (3-5 days) according to the degree of dyspnea and chest imaging progress. The recommended dose could not exceed the equivalent methylprednisolone dosage of 1-2mg/ kg/d (2,24,25), monitoring the blood glucose and controlling by insulin at the same time. In this study, the use of glucocorticoids in patients with diabetes did not affect the prognosis.

The present study has some limitations. First, the number of patients in the present study was limited, and we need a larger sample to verify our conclusion. Second, due to the retrospective analysis, there was the problem of incomplete case information. Finally, the present study included only severe cases and lacked comparisons with mild patients.

Conclusions

This study showed that diabetes, age, neutrophil count and lymphocyte count, blood glucose were independent prognostic factors in patients with COVID-19, but lack of materialization for clinical application. Through further analyzed the prognosis in diabetic patients with severe

COVID-19, it was found that lymphocyte count ≤0.45×10^9 /L, LDH >600 U/L, hsCRP >90 mg/L and IL-10 >10 U/mL were associated with poor prognosis, It is more valuable to judge the condition through the combination of two or more prognostic factors above. People with these characteristics should be taken seriously. Cutoff points were determined and verified to make these prognostic factors specific and increase prognostic value.

Supporting information

S1 Table. Demographics, clinical characteristics and treatment of patients

S2 Table. Cox regression analysis of risk factors for mortality among severe patients

Acknowledgments

We thank all health-care workers involved in the diagnosis and treatment of patients in Wuhan.

Reference

1. Wang D, Hu B, Hu C, et al. Clinical Characteristics of 138 Hospitalized Patients With 2019 Novel Coronavirus-Infected Pneumonia in Wuhan, China. [published online ahead of print, 2020 Feb 7]. JAMA 2020;e201585.

2. Chen N, Zhou M, Dong X, et al. Epidemiological and clinical characteristics of 99 cases of 2019 novel coronavirus pneumonia in Wuhan, China: a descriptive study. Lancet 2020; 395: 507-13.

3. Schoen K, Horvat N, Guerreiro NFC, et al. Spectrum of clinical and radiographic findings in patients with diagnosis of H1N1 and correlation with clinical severity. BMC Infect Dis 2019; 19: 964.

4. National Recommendations for Diagnosis and Treatment of COVID-1019 (the 7th edition). National Health Commission of People's Republic of China. Access 3 Mar 2020. http://www.nhc.gov.cn/xcs/zhengcwj/202003/46c9294a7dfe4cef80dc7f5912eb1989/files/ce3e6945832a438eaae415350a8ce964.pdf.

5. Laboratory diagnostics for novel coronavirus. WHO 2020 (https://www.who.int/health-topics/coronavirus/laboratory-diagnostics-for-novel-coronavirus)(accessed February 6th, 2020).

6. Camp RL, Dolled-Filhart M, Rimm DL. X-tile: a new bio-informatics tool for biomarker assessment and outcome-based cut-point optimization. Clin Cancer Res 2004;10:7252–7259.

7. Gupta R, Ghosh A, Singh AK, et al. Clinical considerations for patients with diabetes in times of COVID-19 epidemic. Diabetes Metab Syndr 2020; 14: 211-212.

8. Hui DS, Azhar EI, Kim Y-J, et al. Middle East respiratory syndrome coronavirus: risk factors and determinants of primary, household, and nosocomial transmission. Lancet Infect Dis 2018; 18: e217-e27.

9. Alqahtani FY, Aleanizy FS, Ali El Hadi Mohamed R, et al. Prevalence of comorbidities in cases of Middle East respiratory syndrome coronavirus: a retrospective study. Epidemiol Infect 2018;147:1 – 5.

10. Arwady MA, Alraddadi B, Basler C, et al. Middle East Respiratory Syndrome Coronavirus Transmission in Extended Family, Saudi Arabia, 2014. Emerg Infect Dis 2016; 22: 1395-402.

11. Yang JK, Feng Y, Yuan MY, et al. Plasma glucose levels and diabetes are independent predictors for mortality and morbidity in patients with SARS. Diabet Med 2006;23:623–628.

12. Zhang JJ, Dong X, Cao YY, et al. Clinical characteristics of 140 patients infected with

SARS-CoV-2 in Wuhan, China [published online ahead of print, 2020 Feb 19]. Allergy 2020;10.1111/all.14238.

13. Yang JK, Lin SS, Ji XJ, et al. Binding of SARS coronavirus to its receptor damages islets and causes acute diabetes. Acta Diabetol 2010; 47: 193-199.

14. Sun ML, Yang JM, Sun YP, et al. Inhibitors of RAS Might Be a Good Choice for the Therapy of COVID-19 Pneumonia. Zhonghua Jie He He Hu Xi Za Zhi 2020;43:E014.

15. Xu X, Chen P, Wang J, et al. Evolution of the novel coronavirus from the ongoing Wuhan outbreak and modeling of its spike protein for risk of human transmission. Sci China Life Sci 2020;63:457–460.

16. Li XC, Zhang J, Zhuo JL. The vasoprotective axes of the renin-angiotensin system: Physiological relevance and therapeutic implications in cardiovascular, hypertensive and kidney diseases. Pharmacol Res 2017; 125: 21-38.

17. Fang L, Karakiulakis G, Roth M. Are patients with hypertension and diabetes mellitus at increased risk for COVID-19 infection? [published online ahead of print, 2020 Mar 11]. Lancet Respir Med 2020.

18. Wong CK, Lam CW, Wu AK, et al. Plasma inflammatory cytokines and chemokines in severe acute respiratory syndrome. Clin Exp Immunol 2004; 136: 95-103.

19. Xia C, Rao X, Zhong J. Role of T Lymphocytes in Type 2 Diabetes and Diabetes-Associated Inflammation. J Diabetes Res 2017; 2017: 6494795.

20. Rubenfeld GD, Caldwell E, Peabody E, et al. Incidence and outcomes of acute lung injury. N Engl J Med 2005;353:1685–1693.

21. Wong CK, Lam CW, Wu AK, et al. Plasma inflammatory cytokines and chemokines in

severe acute respiratory syndrome. Clin Exp Immunol 2004;136:95–103.

22. Conti P , Ronconi G , Caraffa A , et al. Induction of pro-inflammatory cytokines (IL-1 and IL-6) and lung inflammation by COVID-19: anti-inflammatory strategies. [published online ahead of print, 2020 Mar 14]. J Biol Regul Homeost Agents 2020;34:1.

23. Shaw AC, Goldstein DR, Montgomery RR. Age-dependent dysregulation of innate immunity. Nat Rev Immunol 2013; 13: 875-87.

24. Deng SQ, Peng HJ. Characteristics of and Public Health Responses to the Coronavirus Disease 2019 Outbreak in China. J Clin Med 2020;9:575.

25. Tisoncik JR, Korth MJ, Simmons CP, et al. Into the eye of the cytokine storm. Microbiol Mol Biol Rev 2012; 76: 16-32.

Figure legend

Fig 1. Laboratory markers obtained between admission and at discharge or death among survivors and nonsurvivors. The figure shows the temporal changes in lymphocytes (A), interleukin-2 receptor (B), interleukin-6 (C), hsCRP (D), lactate dehydrogenase (E), and serum ferritin (F). The differences between survivors and nonsurvivors were significant at admission and at discharge or death. hsCRP= hypersensitive C-reactive protein.

Fig 2. Overall survival (OS) of severe patients with COVID-19. The y-axis represents the OS rate. Data were analyzed by the Kaplan-Meier method. (A) The OS of diabetic patients and nondiabetic patients ($\chi2=14.405$, $P<0.001$). (B) The OS of five groups by abnormal laboratory indicators in different degrees ($\chi2=58.62$, $P<0.001$). When laboratory indicators exceed the

exceptions listed below, they are included in our groups, including lymphocyte count ≤ 0.45×10^{9} /L, CRP > 90 mg/L, LDH > 600 U/L, IL-10 >10 U/mL . In group a, none of the above four indicators were abnormal; in group b, one indicator was abnormal; in group c, two indicators were abnormal; in group d, three indicators were abnormal; and in group e, four indicators were abnormal.

Figure

Click here to access/download;Figure;Fig 1.tif

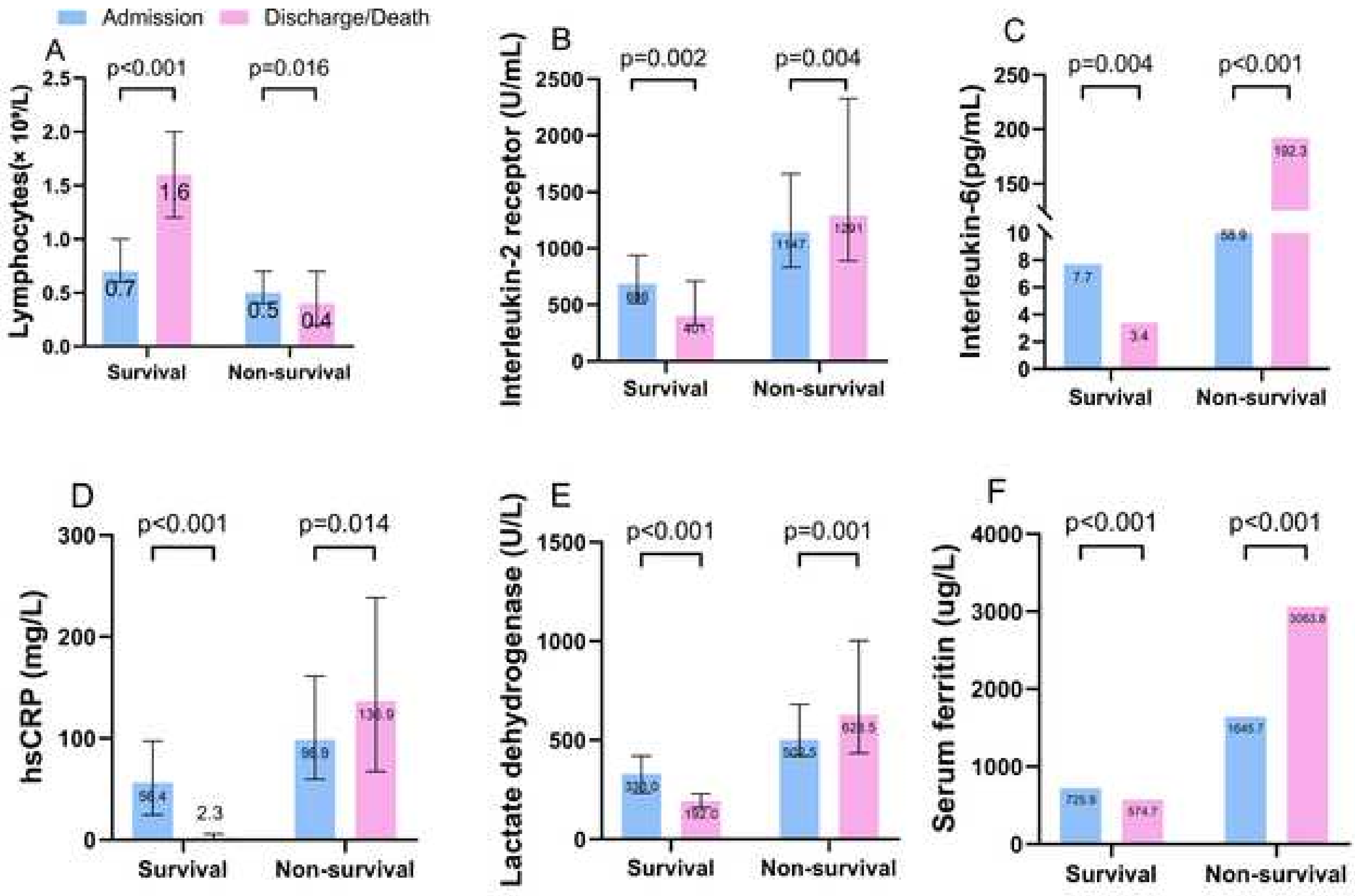

Click here to access/download;Figure;Fig 2.tif

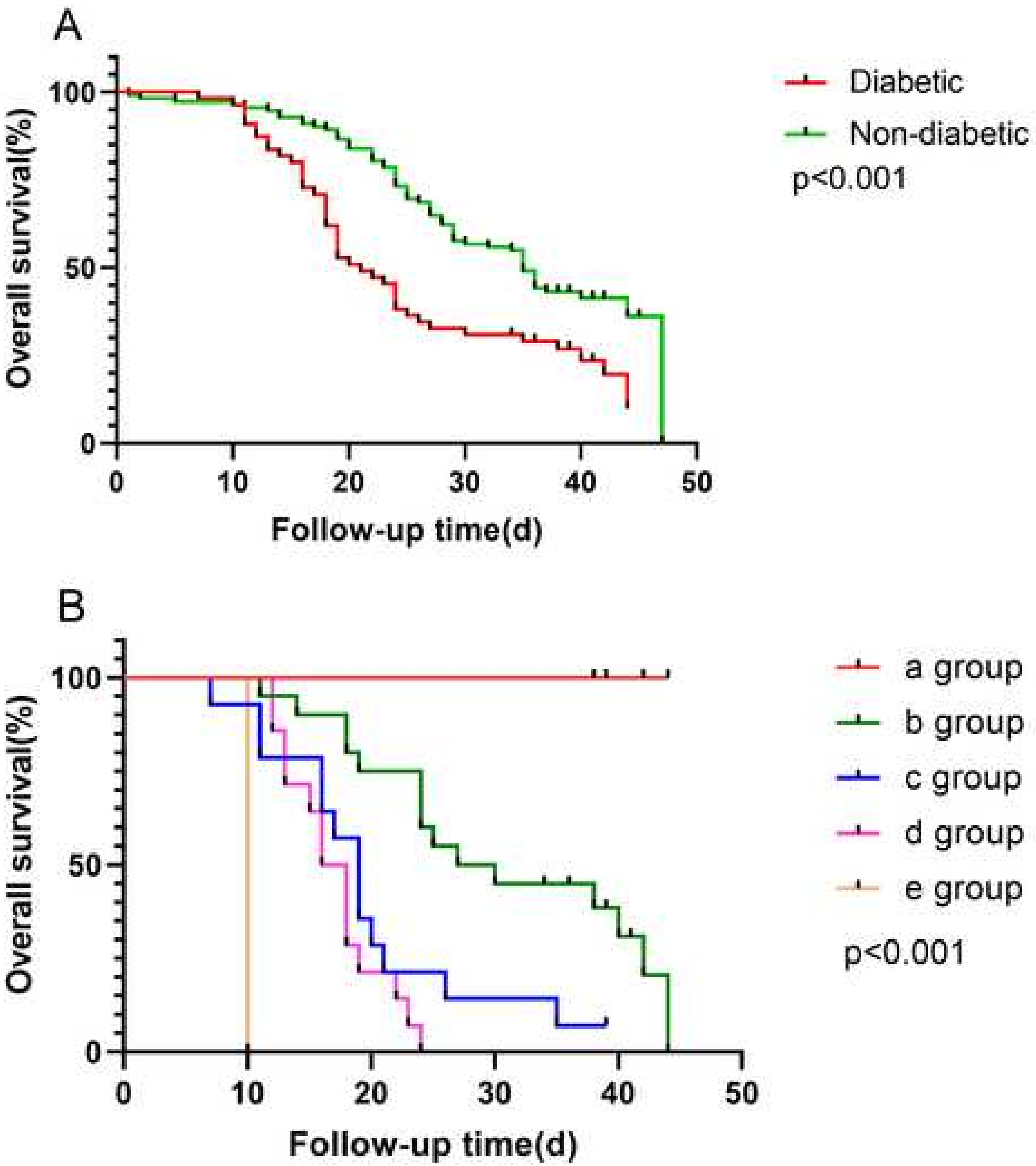

Click here to access/download
Supporting Information
S1 Table.docx

Click here to access/download
Supporting Information
S2 Table.docx
