# Supplementary material for: The risk factors for mortality of diabetic patients with severe COVID-19: A retrospective study of 167 severe COVID-19 cases in Wuhan
Source: PLoS One. 2020 Dec 31;15(12):e0243602. doi: 10.1371/journal.pone.0243602 (PMC7774835; doi:10.1371/journal.pone.0243602)
Supplement: Attachment — Submitted filename: Response to Reviewers.docx [file pone.0243602.s007.docx]

**Author’s Response to the Editor and Reviewers**

**Title:** The risk factors for mortality of diabetic patients with severe COVID-19: a retrospective study of 167 severe COVID-19 cases in Wuhan

**ID: PONE-D-20-21885**

**Version:** 1

**Date:** October 21^th^, 2020

**Corresponding authors:** Donghua Zhang (zdh_62@126.com)

Dear Editor,

We sincerely appreciate all the comments and suggestions of the reviewers and editor to strengthen the manuscript and felt encouraged by your positive feedback. We have extensively revised our paper according to those comments and suggestions, and the changes with track changes in word program are in our revised manuscript. We hope that our efforts have adequately addressed your concerns. Below, we have provided a point-by-point response to the comments.

We look forward to your response.

Sincerely yours,

Donghua Zhang, MD, Ph.D

Department of Hematology, Tongji Hospital

Tongji Medical College

Huazhong University of Science and Technology

1095 Jie-Fang Avenue, Wuhan

Hubei, 430030, P. R. China

Phone: 0086-27-83662830

**Point-by-Point Response to the Reviewers**
**Reviewer 1:**

1. **Response to comment:** There are few typo and grammatical errors.

**Response:** We appreciate the suggestion of the reviewer. We have revised those typo and grammatical errors for better understanding according to the reviewer.

2. **Response to comment:** The discussion should be modified with the two articles that I recommend by comparing them with your results. See the corrections in the PDF attachment.

**Response:** We sincerely appreciate the reviewer's comment. We have carefully read the two articles you have recommend and we modified the discussion by comparing the two articles with our results. We sincerely thank you for the recommendation and corrections in the PDF attachment, which helped to improve the manuscript greatly. Thank you again.

**Reviewer 2**

1. **Response to comment:** Were the 167 patients with severe COVID-19 all of the patients with that condition seen between January and March or were some excluded? On page 6 it is mentioned that “we included 167 patients with complete data…” If some patients were excluded due to incomplete data, the methods of how this was decided should be outlined.

**Response:** We sincerely appreciate the reviewer's comment. All of the patients with severe COVID-19 with that condition were seen between January and March in our hospital, and no patients were excluded. On page 6, "we included 167 patients with complete data...". We mentioned this sentence to emphasize that our data are complete. In fact, information of all the inpatients were recorded in detail, and none of them was excluded because the complete data. This sentence might cause misunderstanding, we have modified it for a better understanding. Thank you.

2. **Response to comment:** Additionally, the specific criteria used to classify patients as “severe” COVID-19 should be outlined as reference 4 is in Chinese and would not be readable by most of the readership of PLOS One. If these criteria are different than what is currently used as an international standard these differences and their implications should be summarized as well.

**Response:** We sincerely appreciate the reviewer's comment. The diagnostic criteria mentioned in reference 4 was the only criteria for diagnostic classification in China during early stage of the pandemic and was also cited by some published articles (Yin L, et al. PLoS ONE, 2020; Fei Z, et al. Lancet, 2020). Internationally, the criteria of ARDS and the diagnostic criteria of sepsis and septic shock can be used to define severe COVID-19 patients (Cascella M, et al. Features, Evaluation, and Treatment of Coronavirus (COVID-19). In: StatPearls. 2020). It requires clinical and ventilatory criteria which is suggestive of a serious new-onset respiratory failure and different degrees of hypoxia. Actually, respiratory failure and hypoxia is just the point that was focused on by the criteria in our manuscript. Therefore, the criteria cited in our manuscript are consistent with international standard. For the convenience of understanding by most of the readership of PLOS One, we have translated the diagnostic criteria into English and cited the criteria of ARDS in the Method part of the revised manuscript.

3. **Response to comment:** Figure 1 shows differences between laboratory values at admission and discharge/death, but these data at the time of discharge/death do not appear to be presented elsewhere in tables or analysis. The study otherwise appears focused on laboratory values on admission. Was a comparison of these makers performed between patients with and without diabetes who died/survived?

**Response:** We sincerely appreciate the reviewer's comment. The data in Figure 1 has been presented in S3 Table as supplementary material. A comparison of these markers performed between patients with and without diabetes who died/survived has been showed in S4 Table as supplementary material. Thanks a lot for your advices.

**4. Response to comment:** Was any analysis performed regarding the degree of glycemic control during admission for patients with diabetes? This would be a very clinically relevant comparison and of interest to readers. If the focus is risk determined solely by laboratory values on admission, that is a reasonable analysis, but it should be more clearly stated.

**Response:** We appreciate the reviewer for the suggestion. This is an important question. We have reviewed data regarding the degree of glycemic control during admission for patients with diabetes from the electronic medical records. The definition of poorly controlled and well controlled hyperglycemia was based on the published criteria (Bruce et al. Glycemic Characteristics and Clinical Outcomes of COVID-19 Patients Hospitalized in the United States, Journal of Diabetes Science and Technology, 2020). We found that the poorly controlled hyperglycemia was associated with poor prognosis in diabetic patients in univariate regression analysis, but showed no significance in multivariate analysis. Moreover, diabetic patients with poorly controlled hyperglycemia had shorter OS than patients with well controlled hyperglycemia. We added these data in Table 1-3 and added a new figure as Fig 2B in in revised manuscript. Therefore, risks to poor prognosis in severe patients with COVID-19 were mainly determined by laboratory values on admission according to our conclusion in this manuscript.

**5. Response to comment:** The values for interleukin-1beta in the tables are all listed as 5, I suspect this was entered in error.

**Response:** We sincerely appreciate the reviewer's carefulness. In our hospital, if the patient's interleukin-1beta level is lower than 5 pg/mL (normal range: 0.0–5.0 pg/mL), it will present as “<5 pg/mL”, which just shows normal values. Actually, the values for interleukin-1beta in the tables are all listed as 5 because interleukin-1beta of most patients were normal. In the table, median and IQR were used to represent this continuous variable, so it was shown as 5.0 (5.0-5.0). Thank you very much for raising this question. We will make an explanation at the bottom of the table for readers' understanding.
